# Bile Acid Signaling Pathways from the Enterohepatic Circulation to the Central Nervous System

**DOI:** 10.3389/fnins.2017.00617

**Published:** 2017-11-07

**Authors:** Kim L. Mertens, Andries Kalsbeek, Maarten R. Soeters, Hannah M. Eggink

**Affiliations:** ^1^Master's Program in Biomedical Sciences, University of Amsterdam, Amsterdam, Netherlands; ^2^Department of Endocrinology and Metabolism, Academic Medical Center, University of Amsterdam, Amsterdam, Netherlands; ^3^Laboratory of Endocrinology, Department Clinical Chemistry, Academic Medical Centre, University of Amsterdam, Amsterdam, Netherlands; ^4^Department of Hypothalamic Integration Mechanisms, Netherlands Institute for Neuroscience, Amsterdam, Netherlands

**Keywords:** bile acids, CNS, brain, FXR, FGF19, TGR5, GLP-1

## Abstract

Bile acids are best known as detergents involved in the digestion of lipids. In addition, new data in the last decade have shown that bile acids also function as gut hormones capable of influencing metabolic processes via receptors such as FXR (farnesoid X receptor) and TGR5 (Takeda G protein-coupled receptor 5). These effects of bile acids are not restricted to the gastrointestinal tract, but can affect different tissues throughout the organism. It is still unclear whether these effects also involve signaling of bile acids to the central nervous system (CNS). Bile acid signaling to the CNS encompasses both direct and indirect pathways. Bile acids can act directly in the brain via central FXR and TGR5 signaling. In addition, there are two indirect pathways that involve intermediate agents released upon interaction with bile acids receptors in the gut. Activation of intestinal FXR and TGR5 receptors can result in the release of fibroblast growth factor 19 (FGF19) and glucagon-like peptide 1 (GLP-1), both capable of signaling to the CNS. We conclude that when plasma bile acids levels are high all three pathways may contribute in signal transmission to the CNS. However, under normal physiological circumstances, the indirect pathway involving GLP-1 may evoke the most substantial effect in the brain.

## Introduction

Bile acids are synthesized in the liver from cholesterol and released in the intestinal lumen upon food intake. They are predominantly known for their role as nutritional detergents that dissolve lipids and lipid-soluble vitamins. However, a growing body of recent literature describes bile acids as versatile signaling molecules (Houten et al., 2006; de Aguiar Vallim et al., 2013; Kuipers et al., 2014), with a widespread distribution of bile acid receptors throughout the organism. Via these receptors, bile acids are capable of modulating their own synthesis (Chiang, 2009; Lefebvre et al., 2009), lipid, glucose and energy metabolism (Thomas et al., 2008a,b; Lefebvre et al., 2009; Schonewille et al., 2016). In addition, bile acids can signal via intermediate signaling molecules that are released upon activation of bile acid receptors in the intestine. The receptors receptive for these intermediate molecules are also distributed ubiquitously throughout the body.

Bile acids and their associated receptors have been detected in the human and rodent brain (Mano et al., 2004a; Ferdinandusse et al., 2009; Keitel et al., 2010; Huang et al., 2016; McMillin et al., 2016; Zheng et al., 2016), however, it is still not clear whether bile acids are capable of signaling to the central nervous system (CNS) and what this signaling could imply. Two recent reviews discussed the role of bile acids in neurological diseases (Ackerman and Gerhard, 2016; McMillin and DeMorrow, 2016), but did not elaborate on the possible physiological effects of bile acid signaling. Therefore, in this review we discuss the signaling pathways of bile acids implicated in the control of energy metabolism under normal physiological circumstances, involving both direct and indirect pathways to the CNS.

## Bile acid metabolism and the enterohepatic circulation

**Figure 1:**
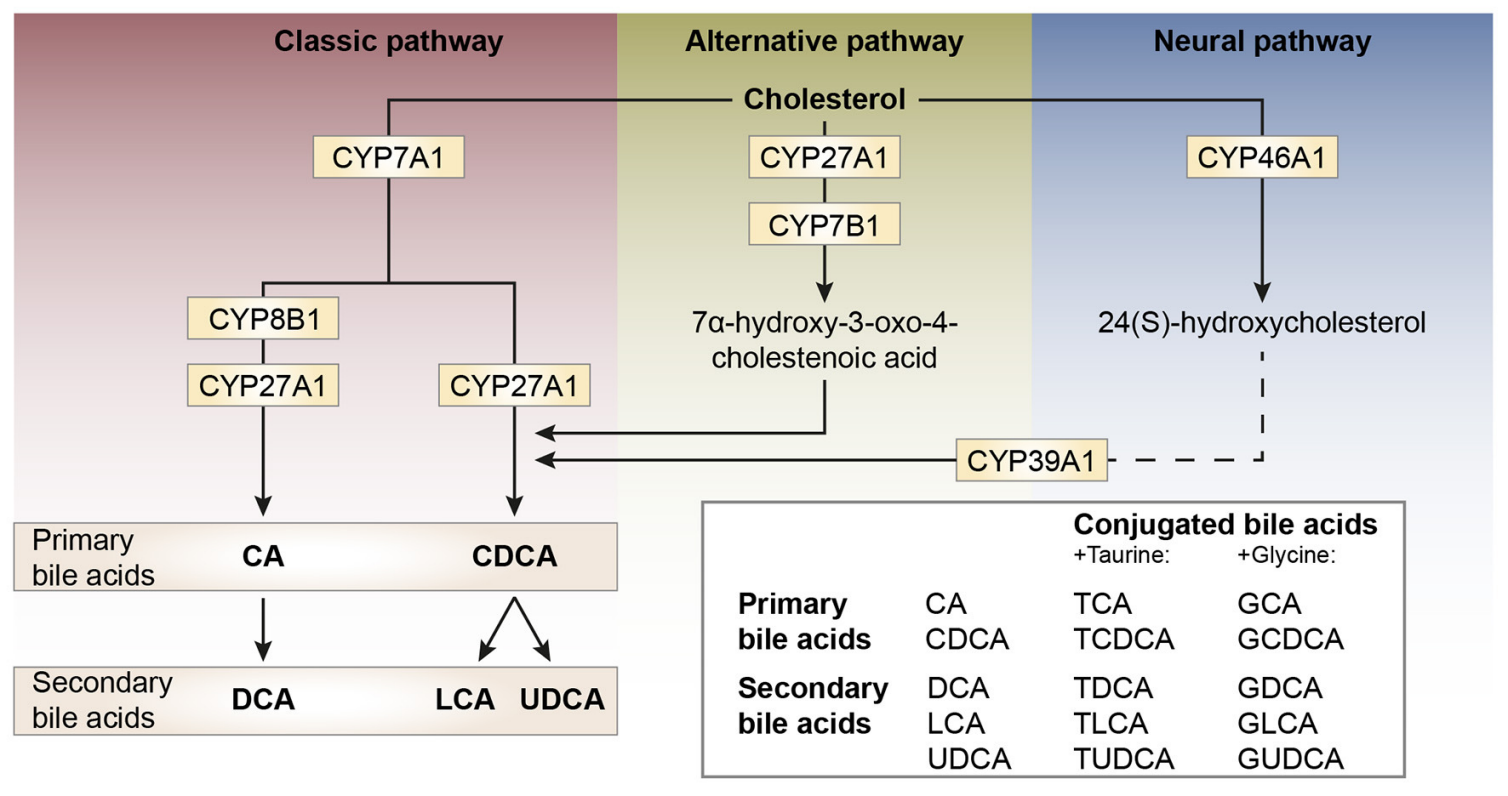
Schematic representation of bile acid synthesis pathways in humans. Bile acid synthesis from cholesterol occurs via different pathways. The classic pathway occurs in the liver and is responsible for the majority of bile acid synthesis. This pathway is initiated by the enzyme cholesterol 7α-hydroxylase (encoded by CYP7A1) and results in the formation of the primary bile acids cholic acid (CA) and chenodeoxycholic acid (CDCA). Key enzymes for the formation of CA or CDCA are sterol 12α-hydroxylase (CYP8B1) and sterol-27 hydroxylase (CYP27A1), respectively. In rodents, the primary bile acids formed are CA and muricholic acid (MCA). The primary bile acids are conjugated to the amino acids glycine (G, mainly in humans) or taurine (T, mainly in rodents) forming conjugated bile acids and bile salts. The formation of secondary bile acids occurs in the intestine under the control of gut flora and when returned to the liver these secondary bile acids can also be conjugated to glycine and taurine. The alternative pathway of bile acid synthesis also occurs in other tissues besides the liver. This pathway is initiated by CYP27A1 and also involves CYP7B1. After several metabolic steps CDCA is formed. The last pathway occurs in the brain and is believed to be important for neuronal cholesterol clearance. Cholesterol is converted to 24(S)-hydroxycholesterol by CYP46A1 and subsequently exits the brain and enters the bloodstream (dotted line). In the liver, bile acid synthesis continues involving CYP39A1 resulting in CDCA after several steps.

**Figure 2:**
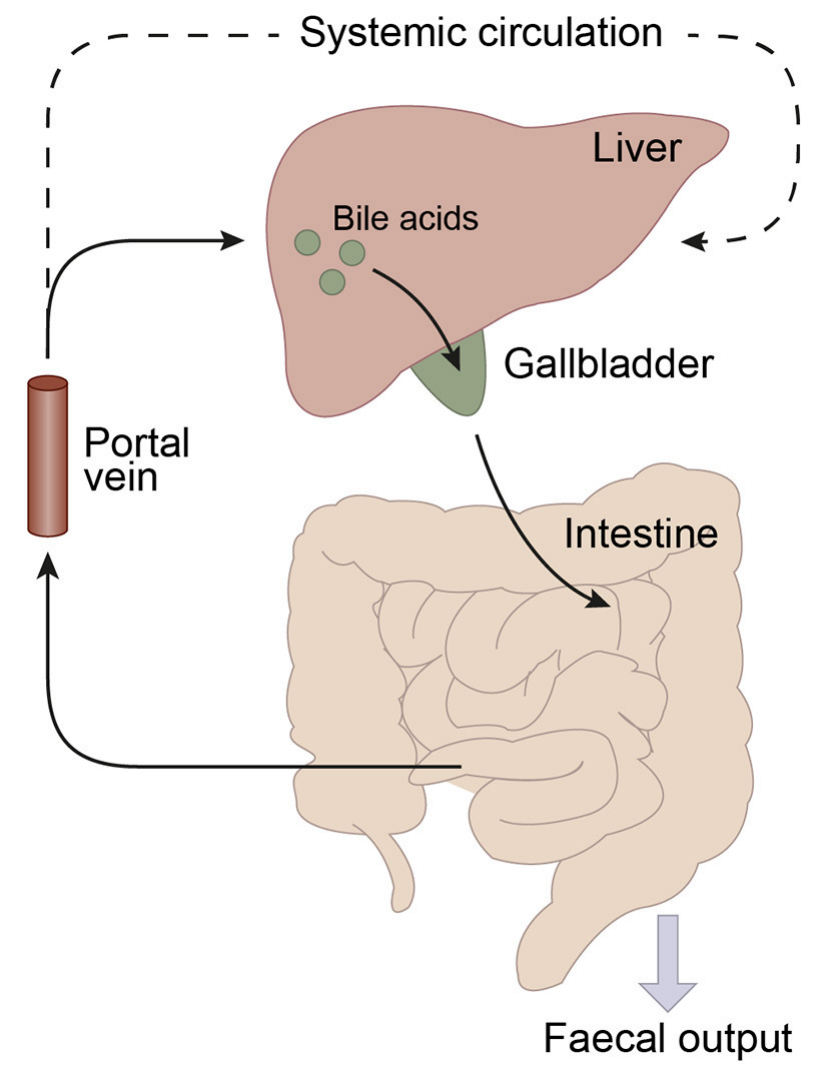
Schematic representation of the enterohepatic circulation of bile acids. Bile acids are synthesized in the liver and stored in the gallbladder. Following food intake, bile acids are released into the duodenum. Traveling down the intestine, the majority of bile acids are taken up by enterocytes. In the jejunum and colon passive diffusion of unconjugated and uncharged bile acids takes place and the ileum is the main site for active uptake of conjugated bile acids by bile salt transporters. About 95% of the bile acids are reabsorbed in the ileum and consequently only a small portion (~5%) of the bile acids is lost through fecal output. The bile acids that are absorbed by the enterocytes are released into the portal vein and redirected to the liver for recycling. Only a small portion escapes the enterohepatic circulation and reaches the systemic circulation. The liver extracts 80–90% of the portal total bile acids.

Bile acid synthesis and enterohepatic cycling have been elaborately reviewed previously (Russell, 2003; Thomas et al., 2008b). In short, bile acids have a cholesterol backbone. Bile acid biosynthesis mainly occurs in hepatocytes (Figure 1), where the classical pathway is initiated by cholesterol 7α-hydroxylase (CYP7A1) which is regulated by the farnesoid X receptor (FXR). The alternative pathway can be initiated by different enzymes that are also expressed outside the liver. *De novo* synthesized bile acids are called primary bile acids. In humans the primary bile acids are cholic acid (CA) and chenodeoxycholic acid (CDCA); in mice the dominant bile acids are CA and muricholic acid (MCA). Subsequently, these bile acids are conjugated with the amino acids glycine (mainly in humans) or taurine (mainly in mice). Bile acids are transported from the hepatocytes through the bile canaliculi and stored, together with cholesterol and phospholipids, in the gallbladder. Following food intake, the presence of nutrients (especially fats and proteins) in the stomach triggers gallbladder emptying which results in the release of bile acids into the duodenum. When bile acids pass through the intestinal tract, they contribute to the absorption of lipids and fat-soluble vitamins. In the intestine, gut microbiota deconjugate and dehydroxylate the primary bile acids, converting them into secondary bile acids and enhancing the diversity of the bile acid pool (Figure 1). In the jejunum and colon, unconjugated, and uncharged bile acids enter the enterocytes through passive diffusion (Figure 2). In the ileum, active uptake of conjugated bile acids takes place by the apical sodium-dependent bile acid transporter (ASBT). In total about 95% of the bile acids are reabsorbed into intestinal enterocytes. The remaining 5% is excreted via feces, a loss which is compensated for by *de novo* bile acid synthesis in the liver. Specific transporters in enterocytes make sure that bile acids are redirected to the liver via the portal vein. In the liver, about ~90% of the bile acids are cleared from the hepatic circulation for reuse. Bile acids can be recycled 4–12 times per day between hepatocytes in the liver and enterocytes in the intestine—which is called the enterohepatic circulation (Mok et al., 1977; Figure 2). Only a small portion (< 10%) of the total bile acid pool reaches the systemic circulation. Systemic plasma bile acid concentrations show a postprandial increase, resulting in a daily rhythm associated with food intake that fluctuates between 5 and 15 μM in humans (Angelin and Bjorkhem, 1977; LaRusso et al., 1978; Schalm et al., 1978; Glicksman et al., 2010; Steiner et al., 2011; Sonne et al., 2016). Also in rodents a daily rhythm of plasma bile acid levels has been reported (Ho, 1976a,b; Zhang et al., 2011; Eggink et al., 2017). These feeding-induced changes indicate that circulating bile acids could provide a postprandial signal, transmitting information about the arrival of nutrients and the subsequent availability of energy (Thomas et al., 2008a). In addition, hepatocytes are equipped with a machinery that can actively promote bile acid excretion when hepatic bile acid concentration increase extensively, as accumulating bile acids can be toxic due to their detergent-like function (Zollner et al., 2006). Consequently, many cases of liver failure or liver damage result in an increased efflux of bile acids into the systemic circulation, leading to high levels of plasma bile acids (Neale et al., 1971; Engelking et al., 1980; Benyoub et al., 2011; Tanaka et al., 2012; Quinn et al., 2014; McMillin et al., 2016).

## Bile acids and the blood-brain barrier

Once in the systemic circulation, bile acids reach the brain via the internal carotid and vertebral arteries that join in an artery ring at the base of the brain—the circle of Willis. From here the arteries arise that ensure blood supply to the brain. In contrast with other capillaries throughout the body, brain capillary endothelial cells are interconnected by tight junctions so substances in the blood need to cross the endothelial cell membranes in order to enter the brain. This blood-brain barrier (BBB) protects the brain from potentially harmful circulating molecules (Bernacki et al., 2008; Abbott et al., 2010).

**Figure 3:**
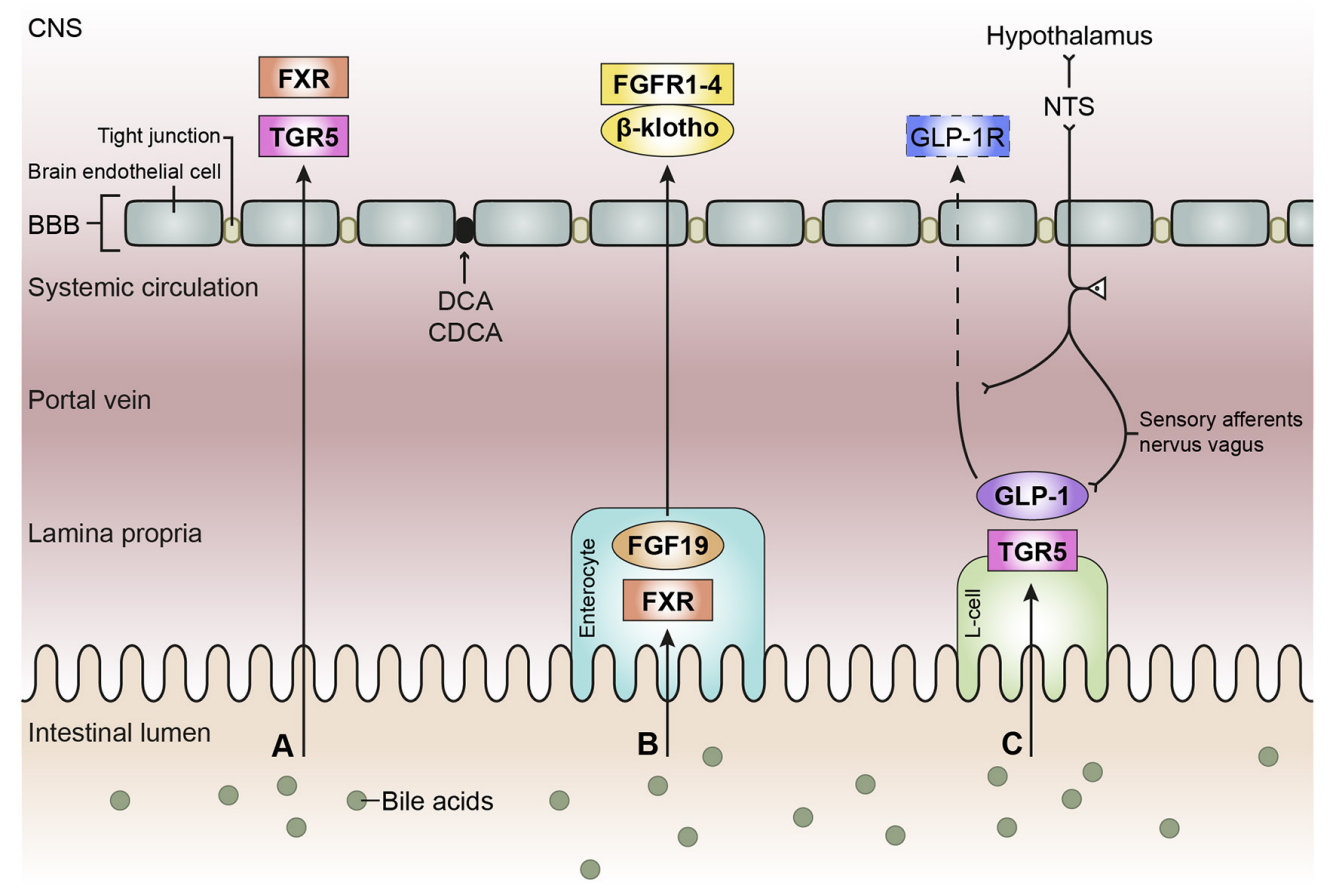
Schematic overview of the bile acid signaling pathways to the central nervous system. Bile acids in the intestinal lumen can signal to the central nervous system (CNS) via different pathways, in this review we focused on the direct pathway **(A)**, the indirect pathway via farnesoid X receptor-fibroblast growth factor 19 (FXR-FGF19) signaling **(B)**, and the indirect pathway via Takeda G protein-coupled receptor-glucagon-like peptide-1 (TGR5-GLP-1) signaling **(C)**. **(A)** Bile acids in the intestine escape the enterohepatic circulation and reach the systemic circulation. Bile acids need to pass the blood-brain barrier (BBB) in order to interact with receptors in the brain, e.g., FXR and TGR5. Deoxycholic acid (DCA) and chenodeoxy cholic acid (CDCA) have been found to interact with gap junction proteins, resulting in a leaky BBB. **(B)** Bile acids taken up by enterocytes can activate the nuclear receptor FXR, which results in the release of FGF19. FGF19 is released by the enterocyte and reaches the portal vein, a small portion of FGF19 will not be taken up by the liver and enters the systemic circulation. FGF19 needs to cross the BBB to interact with FGF receptors (1–4) in the brain. The protein β-klotho is necessary for the formation of a stable receptor-complex. **(C)** in the intestine, a specific group of enteroendocrine cells, L-cells, produces GLP-1 upon the activation of TGR5 which can be triggered by bile acids. GLP-1 is quickly degraded by the enzyme dipeptidyl peptidase-4 (DPP-4, not shown), consequently high concentrations of GLP-1 are only found in the lamina propria of the intestine. A small portion of intact GLP-1 reaches the portal vein and even a smaller portion reaches the systemic circulation. It is questionable whether sufficient intact GLP-1 reaches the brain to interact with GLP-1 receptors, hence the dashed line. GLP-1 receptors are also expressed on afferent terminals of the vagal nerve present in the lamina propria and portal vein. The vagal nerve projects to the nucleus of the solitary tract (NTS) in the brainstem, from where projections are directed toward other brain regions, e.g. the hypothalamus (the vagal-brainstem-hypothalamic pathway).

There are reports that both unconjugated and conjugated bile acids can cross the BBB (Keene et al., 2001; Palmela et al., 2015; McMillin et al., 2016; Figure 3A), however, the involved mechanisms are still uncertain. Unconjugated bile acids might diffuse across the BBB, because CA, CDCA, and deoxycholic acid (DCA) are capable of diffusing across phospholipid bilayers (Kamp and Hamilton, 1993) and their brain levels correlate with their serum levels (Higashi et al., 2017). Indeed, unconjugated ursodeoxycholic acid (UDCA) crossed the BBB in a dose depend manner in orally treated amyotrophic lateral sclerosis patients (Parry et al., 2010). Conjugated bile acids need active transport to cross the BBB due to their larger structure and amphipathic nature (St-Pierre et al., 2001). Indeed, several xenobiotic and bile acid transporters found in the liver, intestine, and kidney are also present at the BBB and choroid plexus providing the machinery for bile acid transport over the BBB. These include members of the solute carrier (SLC) family such as the organic anion transporting polypeptides (OATP) and ASBT, and members of the ATP-binding cassette transporters (ABC) family such as the multidrug resistance protein (MRD) 2 and 4 (Choudhuri et al., 2003; Bernacki et al., 2008; Abbott et al., 2010; Klaassen and Aleksunes, 2010; Ballatori, 2011; Table 1). The main function of these transporters is to protect the brain from potentially toxic molecules by transporting them out of the brain into the bloodstream (Abbott et al., 2010). However, the presence of these transporters on both the basolateral (blood-facing) and apical (brain-facing) side, also facilitates the transport of molecules from the systemic circulation into the CNS (Abe et al., 1998; Klaassen and Aleksunes, 2010). Of interest, an *in situ* rat brain perfusion with [^3^H]TCA resulted in no significant uptake of the bile acid in the ipsilateral hemisphere within 2 min, suggesting that the labeled TCA did not cross the BBB (Kitazawa et al., 1998). Direct evidence of *in vivo* transport of bile acids over the BBB via their transporters is still lacking.

**Table 1:** Bile acid transporters found in the brain.

**Bile acid transporter**	**Localization**	**References**	**Function and substrate forms**
MRP2	Apical/Basolateral Brain endothelial cells in mouse and rat	Miller et al., 2000; Soontornmalai et al., 2006,^1^	The ABC family are active efflux pumps that transport chemicals through a membrane. Substrates include: TCA and its sulfated forms.
MRP3	Tight junction of Choroid Plexus in mouse	Soontornmalai et al., 2006,^1^	Substrates include: preferably conjugated bile acids.
MRP4	Apical Brain Capillary endothelial cells in rat and human	Nies et al., 2004; Roberts et al., 2008,^1^	Substrates include: CA and conjugated bile acids.
OSTα/OSTβ	No reports on mRNA or protein in brain, minimal mRNA found in mice	Klaassen and Aleksunes, 2010; Ballatori, 2011	In the intestine, OSTα/β transports bile acids across the basolateral membrane of the enterocyte: releasing them to the portal vein.
BSEP	mRNA expression in rat choroid plexus is ~2.75% of hepatic Bsep expression	Choudhuri et al., 2003	In the liver, BSEP is an active transport mechanism across the canalicular membrane of the hepatocyte: secreting conjugated bile acids into the bile ducts.
OATP1A1	Apical Choroid plexus epithelial cells in rat	Angeletti et al., 1997,^1^	The SLC family are typical uptake transporters even though some can function bidirectional. Substrates include: unconjugated and conjugated bile acids.
OATP1A4	Basolateral Choroid plexus epithelial cells in rat; mRNA in brains of male and female C57BL/6 mice	Gao et al., 1999; Cheng et al., 2005,^1^	Substrates include: unconjugated and conjugated bile acids.
OATP1A2	In brain capillary endothelial cells in human, but not determined what side	Lee et al., 2005,^1^	Substrates include: unconjugated and conjugated bile acids
OAT3	Basolateral Brain Capillary endothelial cells in rat	Kikuchi et al., 2003; Roberts et al., 2008,^1^	Substrates include: unconjugated and conjugated CA.
NTCP	mRNA expression in rat choroid plexus is ~1.8% of hepatic Ntcp expression	Choudhuri et al., 2003	In the liver, NTCP transports bile acids across the basolateral membrane of the hepatocyte in a sodium-dependent manner: facilitating uptake of unconjugated and conjugated bile acids from the portal blood.
ASBT	Rat and mouse hypothalamus and frontal cortex, low mRNA expression in human brain and rat choroid plexus	Choudhuri et al., 2003; Klaassen and Aleksunes, 2010; McMillin et al., 2015; Nizamutdinov et al., 2017	In the intestine, ASBT transports bile acids across the apical membrane of ileal enterocytes in a sodium-dependent manner: absorption of unconjugated and conjugated bile acids from the intestine. ASBT on the apical surface of cholangiocytes participate in the cholehepatic recirculation. ASBT in the brain could facilitate uptake of bile acids into neurons and other brain cells. Subsequently, intracellular bile acids could activate nuclear receptor.

1 *References are according to Klaassen and Aleksunes (2010). ABC, ATP-binding cassette transporters; MRP, multidrug resistant protein; OST, organic solute transporter; BSEP, bile salt export pump; SLC, solute carriers; OATP, organic anion transporting polypeptide; OAT, organic anion transporter; NTCP, Na^+^ taurocholate cotransporting polypeptide; ASBT, apical sodium-dependent bile acid*.

### Plasma bile acid levels and the integrity of the blood-brain barrier

During liver failure, plasma bile acid levels can increase dramatically (Benyoub et al., 2011; Tanaka et al., 2012; Quinn et al., 2014; McMillin et al., 2016), sometimes even up to 20-fold in rats (Quinn et al., 2014) and 100-fold in humans (Neale et al., 1971; Engelking et al., 1980). At high concentrations (≥1.5 mM) bile acids are capable of damaging the lipid layers of the BBB (Greenwood et al., 1991), due to their detergent and lytic action on cell membranes (Naqvi et al., 1970; Greenwood et al., 1991). At lower concentrations (0.2–1.5 mM), bile acids may modify the BBB in a more subtle way (Greenwood et al., 1991). The bile acids CDCA and DCA increase phosphorylation of the tight junction protein occludin in a Rac1-dependent mechanism, resulting in the disruption of tight junctions (Quinn et al., 2014) and leading to increased permeability of the BBB (Greenwood et al., 1991; Quinn et al., 2014). Consequently, allowing bile acids and other molecules to diffuse into the brain. UDCA and its glycine-conjugated form glyco-ursodeoxycholic acid (GUDCA) exert a protective effect on brain endothelial cells by reducing apoptosis (Palmela et al., 2015). In addition, a recent study showed that microglial cells express TGR5 and that binding of taurine-conjugated UDCA (TUDCA) to TGR5 has anti-inflammatory effects in a mouse model of acute brain inflammation (Yanguas-Casás et al., 2017). This could explain the neuroprotective effects of TUDCA observed as reduced neuronal apoptosis in several animal models for neurodegenerative diseases, such as Huntington's disease (Keene et al., 2001, 2002), Alzheimer's disease (Sola et al., 2006; Viana et al., 2009), Parkinson's disease (Duan et al., 2002), acute ischemia (Rodrigues et al., 2002), and hemorrhagic stroke (Rodrigues et al., 2003). These findings highlight the physiological differences of bile acid species, where DCA and CDCA interfere and disturb gap junction function in the BBB, but UDCA and its conjugated forms exert a protective effect on brain endothelial cells and neurons. It is still unknown whether these different effects are due to the different affinities of these bile acids for FXR.

## Bile acids in the central nervous system

When plasma bile acid concentrations increase during hepatic failure, cerebral bile acid levels also rise excessively in humans and rodents (Bron et al., 1977; Ceryak et al., 1998; Tripodi et al., 2012). Additional reports suggest that these elevated levels of bile acids are derived from the systemic circulation (DeMorrow et al., 2012; Quinn et al., 2014; McMillin et al., 2015, 2016; Palmela et al., 2015). Also in healthy conditions detectable levels of both conjugated and unconjugated bile acids have been reported in the brain, both in rodents and humans (Mano et al., 2004a; Zheng et al., 2016; Higashi et al., 2017; Pan et al., 2017; Table 2). In rats no glycine-conjugated bile acids were detected in the rat brain (Mano et al., 2004a; Higashi et al., 2017). In one study CDCA is the most abundantly present bile acid in the rat brain, making up 92.1% of the total amount of cerebral bile acids and mainly being found in protein-bound form conceivably preventing it from exiting the brain (Mano et al., 2004a). However, a different study found that CA was most abundantly present in the rat brain and did not find the high amounts of protein-bound CDCA (Higashi et al., 2017). The discrepancy in CDCA levels between these studies could not be sufficiently explained. In addition, various bile acid transporters are expressed in the CNS such as ASBT in the hypothalamus and frontal cortex (McMillin et al., 2015; Nizamutdinov et al., 2017; Table 1), providing a mechanism for the neuronal uptake of bile acids.

**Table 2:** Bile acids found in the brain.

**Bile acids**	**Localization**	**References**	**Function and diseased state**
Unconjugated CDCA, DCA, and CA	Adult male and female Wistar rats	Mano et al., 2004a	No clear function. Bile acids were mainly found in protein-bound form.
Unconjugated CDCA, DCA, and CA	Adult male Wistar and Sprague Dawyley rats	Higashi et al., 2017	No clear function. Bile acids were not found in protein-bound form.
CDCA, DCA, LCA; TUDCA, TCA, TCDCA, TαMCA, TβMCA	Adult male wild-type C57BL/6 and FXR KO mice	Huang et al., 2015	Function is unknown but FXR KO mice had higher levels of bile acids in serum and (thus?) in brain.
Total bile acids	Adult Sprague Dawyley rats hypothalamic tissue measured by EIA	McMillin et al., 2015	In a cholestasis model serum bile acids increased and gained entry into the brain via a leaky BBB. Intracellular hypothalamic bile acids may have a role in modulating the HPA axis during liver disease.
C24-bile acids (i.e., the sum of conjugated and unconjugated CA, CDCA, UDCA, DCA)	PDD patients, DBP deficiency patients and control subjects	Ferdinandusse et al., 2009	No difference in total C24-bile acids between patients and controls. Further analysis of different brain areas also showed no differences.
	No bile acids were found in human CSF, only intermediates of bile acid synthesis	Ogundare et al., 2010	
CA, GCA, TCA, CDCA, GCDCA, TCDCA, DCA, GDCA, LCA, UDCA	Neocortex, Brodmann area 7 of AD patients and age-matched controls	Pan et al., 2017	The amount of TCA was significantly lower in AD patients compared to age-matched controls with no form of dementia.
CA, TCA, DCA, LCA, MCA, TMCA, TUDCA	Adult female APP/PS1dE9 and C57BL/6J mice	Pan et al., 2017	The AD model mice had significant lower amounts of brain bile acids.

*CDCA, chenodeoxycholic acid; DCA, deoxycholic acid; CA, cholic acid; LCA, lithocholic acid; TUDCA, taurine conjugated ursodeoxycholic acid; MCA, muricholic acid; EIA, enzyme-linked immunoassay; PDD, peroxisome deficiency disorder; DBP, D-bifunctional protein; AD, Alzheimer Disease*.

The unconjugated bile acids (CA, CDCA, and DCA) seem to be mostly derived from the periphery by passive diffusion as brain levels correlate with serum levels and intraperitoneally injected D_4_-CA and D_4_-CDCA are well-detected in the brain (Higashi et al., 2017). There are also indications that at least parts of the biosynthesis pathway for bile acids is present in the brain, because involved enzymes and intermediates have been detected locally (Cali et al., 1991; Björkhem et al., 1998; Lund et al., 1999; Li-Hawkins et al., 2000; Mano et al., 2004a,b; Ogundare et al., 2010). Since only a part of the biosynthesis pathway of bile acids is present in the brain, its main function has been proposed to be cholesterol clearance (McMillin and DeMorrow, 2016).

## Bile acid receptors in the central nervous system

The most studied bile acid receptors are FXR (Makishima et al., 1999; Parks et al., 1999; Wang et al., 1999) and the Takeda G protein-coupled receptor 5 (TGR5) (Maruyama et al., 2002; Kawamata et al., 2003). Both receptors are abundantly expressed in the enterohepatic circulation, but also in the brain [FXR: (Huang et al., 2016; McMillin et al., 2016); TGR5: (Maruyama et al., 2002, 2006; Keitel et al., 2010; Yanguas-Casás et al., 2017)]. Other receptors that might bind bile acids and can be found in the CNS are summed in Table 3, their possible functions are reviewed elsewhere (McMillin and DeMorrow, 2016).

**Table 3:** Bile acid receptors found in the brain.

**Bile acid receptors**	**Localization**	**References**	**Function and bile acid affinity**
FXR	Human and mouse, mRNA and protein level	Huang et al., 2016; McMillin et al., 2016	The function FXR in the brain is still unclear. FXR KO mice showed disrupted neurotransmitter systems. In an acute liver failure model blockage of central FXR signaling delayed neurological decline. Substrate affinity: CDCA >> DCA, LCA > CA > UDCA, MCA (antagonist).
TGR5	Human and rat, mRNA and protein level	Maruyama et al., 2002; Kawamata et al., 2003; Keitel et al., 2010; Yanguas-Casás et al., 2017	The function of TGR5 in the brain is under investigation. TGR5 is present in various cell types such as neurons, glia and microglia. TGR5 can also be activated by various neurosteroids so TGR5 might also have bile acid independent functions in the brain. Substrate affinity: LCA > DCA> MCA > CDCA > CA > HDCA > UDCA.
PXR	mRNA and protein level in mouse primary hippocamal neurons	Litwa et al., 2016	Xenobiotic nuclear receptor that can activate Cytochrome P450 enzymes to dispose toxins, for example at the BBB. In the brain neuronal PXR is involved in the propagation of the neurotoxic and apoptotic effects of nonylphenol.
VDR	Human brain protein expression of VDR is strikingly similar to rodents	Eyles et al., 2005, 2014	In the brain vitamin D can act as a neurosteroid via the VDR. In de adult rat brain VDR is not localized to the membrane questioning its role in a fast calcium response.
GR	Adult Sprague Dawyley rats	Miura et al., 2001; McMillin et al., 2015	UDCA can bind and translocate the GR. McMillin et al., propose that intraneuronal bile acids in the hypothalamus can activate the GR and subsequently supress the HPA axis.
S1PR2	Developing brain and adult rodent brain	McMillin and DeMorrow, 2016; McMillin et al., 2017	Indirect evidence suggest that S1PR2 functions in the brain and *in vitro* studies show that conjugated bile acids can activate S1PR2.
M3		Raufman et al., 2002, 2003	Muscarine acetylcholine receptors (M1-5) are expressed throughout the CNS. Chinese Hamster Ovary cells that express the M3 can be activated by TLCA *in vitro*, albeit with high concentrations.

*FXR, farnesoid X receptor; TGR5, Takeda G protein-coupled receptor; PXR, pregnane X receptor; VDR, vitamin D receptor; CAR, constitutive adrostane receptor; GR, glucocorticoid receptor; S1PR2, sphingosine 1-phosphate receptor 2; M3, muscarine acetylcholine receptors; CNS, central nervous system*.

## Effects of elevated plasma bile acid levels on the central nervous system

As mentioned above, receptors able to bind bile acids are also expressed in the CNS and thus are capable of mediating the actions of bile acid signaling. Most studies investigated the effects of central bile acid signaling in the pathological state or pharmacologically administered bile acids directly into the brain (reviewed in Ackerman and Gerhard, 2016; McMillin and DeMorrow, 2016). For example a study investigating hepatic encephalopathy induced by acute liver failure in mice found doubled amounts of bile acids in the brain compared to the control situation (McMillin et al., 2016). The elevated plasma and cerebral bile acid levels consequently generate an amplified effect and show what the possible consequences are of pathologic bile acid signaling in the brain. The variety of effects of bile acids in the diseased brain (McMillin and DeMorrow, 2016) illustrates that bile acids cannot be seen as one signal, but different forms have different effects, including their difference in affinity for the receptors. Moreover, they do not reflect on the effects of bile acid signaling to the CNS under normal physiological circumstances caused by the postprandial elevated plasma bile acid levels, of which little is known.

## Indirect bile acid signaling to the central nervous system via FXR-FGF15/19 pathway

In addition to the direct signaling pathway described in the previous section, bile acids can also provide a signal to the CNS via the gut-brain axis. After their release into the intestine, bile acids can interact with receptors in the gastrointestinal system and thereby initiate a signal cascade that reaches the CNS. In this section we will discuss the indirect pathway initiated by FXR activation and the release of fibroblast growth factor (FGF) 15/19 (Figure 3B). FXR is primarily activated by CDCA and CA and to a lesser extent by DCA and LCA (Makishima et al., 1999; Parks et al., 1999; Wang et al., 1999). In contrast, UDCA and muricholic acid (MCA) do not seem to activate FXR (Makishima et al., 1999; Parks et al., 1999; Wang et al., 1999) and even seem to antagonize FXR in mice (Sayin et al., 2013; Hu et al., 2014), highlighting an important difference between humans and mice, because MCA is the major bile acid in mice and does not occur in humans (Takahashi et al., 2016). FXR is extensively expressed in hepatocytes and enterocytes. In the enterohepatic circulation FXR functions as a bile acid sensor, providing negative feedback to the bile acid synthesis and transport machinery when bile acid levels rise. For an extensive overview of FXR function in the enterohepatic circulation we recommend (Lefebvre et al., 2009; De Magalhaes Filho et al., 2017).

### Intestinal FXR and FGF15/19

In the intestine, activation of FXR can trigger the production of FGF19, a FGF with hormonal characteristics (Holt et al., 2003; Potthoff et al., 2012). The rodent orthologue for human FGF19 is FGF15, which has comparable, but not necessarily identical functions (Inagaki et al., 2005; Jones, 2008). The most abundant expression of FGF15/19 is found in the terminal ileum of the intestine (Holt et al., 2003; Inagaki et al., 2005; Fon Tacer et al., 2010). Bile acids absorbed by enterocytes can activate the nuclear receptor FXR, which leads to the production of FGF15/19 (Kliewer and Mangelsdorf, 2015). The enterocytes release FGF15/19 from their basolateral membrane into the portal vein. Subsequently, FGF15/19 activates the fibroblast growth factor receptor (FGFR) 4 in the liver and this leads to the inhibition of *de novo* bile acid synthesis by inhibition of Cyp7a1. Liver and intestinal FXR KO models have shown that *Cyp7a1* inhibition depends mostly on intestinal FXR activation via FGF15 (Kim et al., 2007). FGF19 mRNA is expressed in the intestine and in hepatocytes in the liver, while in mice FGF15 mRNA is only expressed in the intestine (Song et al., 2009; Fon Tacer et al., 2010). Outside the enterohepatic cycle FGF15/19 can signal in an endocrine manner and is involved in lipid and glucose metabolism (Owen et al., 2015). In both human and mouse FGF15/19 mRNA is widely expressed in the developing brain (Nishimura et al., 1999; Ford-Perriss et al., 2001; Gimeno et al., 2003), but not in the adult brain (Nishimura et al., 1999; Fon Tacer et al., 2010).

Plasma levels of FGF15 in mouse (Katafuchi et al., 2015) and FGF19 in humans (Lundåsen et al., 2006) have been found to follow a daily rhythm. In humans, plasma FGF19 levels respond to food intake and bile acids, showing a postprandial increase following the peak of plasma bile acid levels ~3 h after a meal (Lundåsen et al., 2006; Sonne et al., 2016). In contrast, a different study found that FGF19 levels predominantly respond to carbohydrate intake compared to lipid or protein intake and concluded that this would dissociate the FGF19 response from bile acid signaling (Morton et al., 2013a). Which is an important issue for further research.

### FGF15/19 signaling in the central nervous system

FGF19 in the systemic circulation is capable of crossing the BBB and is relatively stable in the brain (Hsuchou et al., 2013). In addition, FGFRs are expressed in the brain (Wanaka et al., 1990; Yazaki et al., 1994; Belluardo et al., 1997; Reuss and von Bohlen und Halbach, 2003), suggesting that FGF19 could signal from the intestine to the CNS. FGF19 binds directly to FGFR4 but a more solid bond is realized when β-Klotho is bound to the FGF19-FGFR4 complex (Xie et al., 1999; Harmer et al., 2004; Wu et al., 2007, 2009). The single-pass transmembrane protein β-Klotho serves as a cofactor for FGF19 activity by physically interacting with FGFRs, increasing the affinity of FGF19 for FGFRs and causing efficient FGF signaling (Kurosu et al., 2007; Ogawa et al., 2007). For successful binding of FGF19 with FGFR1c, 2c, or 3c the presence of β-Klotho is essential (Kurosu et al., 2007; Wu et al., 2009; Yang et al., 2012). Whereas, FGFR1c, 2c, and 3c are highly expressed in the brain, β-Klotho is not and is selectively expressed in particular regions including the suprachiasmatic, arcuate, and paraventricular nucleus of the hypothalamus, the area postrema and solitary nucleus of the dorsal-vagal complex and the nodose ganglia (Bookout et al., 2013; Liang et al., 2014; Owen et al., 2015). These regions also express FGFRs (Fon Tacer et al., 2010; Bookout et al., 2013; Ryan et al., 2013), however, to our knowledge no studies looked at the co-expression of FGFRs and β-Klotho. In the periphery the main target receptor of FGF15/19 is FGFR4. In the brain, the expression of FGFR4 has been detected in the hypothalamus (Ryan et al., 2013) and in cholinergic neurons in the medial habenular nucleus (Itoh et al., 1994; Miyake and Itoh, 1996). Overall the expression of FGFR4 in the CNS is less abundant than FGFR1c-3c (Fon Tacer et al., 2010). Interestingly, intraperitoneal (ip) FGF19 injections in mice resulted in increased FGFR activity in the hypothalamus, more specifically in the arcuate nucleus (ARC) (Marcelin et al., 2014). Staining for pERK1/2 revealed that in the ARC the AGRP/NPY (agouti-related peptide/neuropeptide Y) neurons and not the POMC (pro-opiomelanocortin) neurons were involved in FGF19 signaling. NPY and POMC neurons modulate feeding behavior by stimulating and inhibiting appetite, respectively (van den Heuvel et al., 2011; Gumbs et al., 2016). Intracerebroventricular (icv) administration of FGF19 decreased neural activation in the ARC as measured by c-Fos expression and reduced gene expression of *Agrp* and *Npy* (Marcelin et al., 2014), suggesting that central FGF19 signaling inhibits AGRP/NPY neurons in the ARC.

Taken together, FGF15/19 signaling in the CNS can generate a wide spread of effects via the FGFRs that are present in the hypothalamus, medial habenular nucleus and dorsal-vagal complex. Consequently, bile acids in the enterohepatic circulation can extend their signal to these cerebral regions via the FXR-FGF15/19 pathway.

### Central FGF15/19 improves metabolic rate and glucose metabolism

The effects of central FGF19 are mainly studied in animal models for obesity and diabetics, because overexpression of FGF19 in mice resulted in increased energy expenditure and animals on a high fat diet (HFD) did not become diabetic or obese (Tomlinson et al., 2002). Intravenous (iv) administration of FGF19, also prevented genetic (ob/ob) and diet-induced (HFD) obese mice to develop diabetes by improving glucose metabolism (Fu et al., 2004). This beneficial effect of systemic FGF19 on glucose metabolism was reduced by 50% when an FGFR antagonist was infused in the brain (Morton et al., 2013b). In addition, rats on a HFD showed reduced expression of hypothalamic FGFR1 and 4 compared to chow-fed rats (Ryan et al., 2013). These findings suggest that central FGFR signaling is involved in energy and glucose metabolism.

Icv FGF19 administration increased the metabolic rate in wild type mice (Fu et al., 2004) and in HFD-fed and ob/ob mice reduced weight gain and improved glucose metabolism (Marcelin et al., 2014). A single administration of icv FGF19 had no effect on the energy expenditure, but improved glucose metabolism in ob/ob mice and mice on a HFD (Morton et al., 2013b; Marcelin et al., 2014) as well as in lean and HFD-fed rats (Ryan et al., 2013). Pretreatment with an FGFR inhibitor in the brain blocked the beneficial effects of icv FGF19 on glucose metabolism (Morton et al., 2013b). In a rat model for type 1 diabetes, hyperglycemia could be reversed by icv administration of FGF19 (Perry et al., 2015). An additional observation was that central FGF19 resulted in decreased adrenocorticotropic hormone (ACTH) and corticosterone plasma levels, suggesting the suppression of HPA activity (Perry et al., 2015). The majority of the studies, investigating the effects of central FGF19 on glucose metabolism found no differences in insulin sensitivity or secretion that could explain improved glucose metabolism (Morton et al., 2013b; Ryan et al., 2013; Perry et al., 2015). However, one study did find improved insulin sensitivity in ob/ob and HFD-fed mice treated with icv FGF19 compared to vehicle treated mice (Marcelin et al., 2014). These studies highlight the controversy concerning the involved mechanisms that explain the effects of central FGF19 on glucose metabolism.

Consequently, these studies provide different explanations concerning the mechanisms that drive the beneficial effect of central FGF19 action on glucose and energy metabolism. This highlights that further research is necessary to reveal the underlying mechanisms that mediate central FGF19 action. Altogether, little is known about the neurocircuitry involved in FGF19-FGFR signaling that could be instigated by bile acid binding to FXR in the intestine. In addition, it should be studied to which extend the different FGFs contribute to the FGFRs signaling in the CNS, because FGF21 binds to the same FGFR and β-Klotho complexes as FGF19 and generates similar effects when administered in the brain (Owen et al., 2015; Degirolamo et al., 2016). However, above all the question remains whether the postprandial increase in plasma FGF19 is sufficient to elicit a substantial effect in the CNS.

## Indirect bile acid signaling to central nervous system via TGR5-GLP-1 pathway

In this section we will discuss the indirect pathway involving signaling via intestinal TGR5 (Figure 3C), which is the other well-studied bile acid receptor that is expressed abundantly in the enterohepatic circulation (Thomas et al., 2008a). The TGR5 receptor can be activated by both conjugated and unconjugated bile acids, with litocholic acid (LCA) and taurolitocholic acid (TLCA) being the most potent bile acids (Kawamata et al., 2003). In the brain, TGR5 can also be activated by other endogenous ligands, such as neurosteroids (Keitel et al., 2010). In the intestine, stimulation of TGR5 by bile acids can also result in the release of the gut hormone GLP-1, which is capable of extending the bile acid signal from the intestine to other parts of the body, including the CNS (Figure 3C; Katsuma et al., 2005; Thomas et al., 2009; Ullmer et al., 2013).

### Intestinal TGR5 and GLP-1

GLP-1 is an incretin that influences energy homeostasis by reducing appetite and food intake and inhibiting gastric emptying (Drucker and Nauck, 2006). In the gut a particular group of entero-endocrine cells, L-cells, are responsible for the production of GLP-1 and are predominantly found in the terminal ileum and colon (Drucker and Nauck, 2006; Lim and Brubaker, 2006). The action of bile acids on GLP-1 release is predominantly regulated via TGR5 receptors located at the basolateral membrane of L-cells, thus not facing the lumen of the intestine (Brighton et al., 2015). This means that bile acids first need to cross both the apical and the basolateral membrane of intestinal cells in order to activate TGR5 and provoke a GLP-1 response. L-cell GLP-1 release follows a circadian rhythm suggesting it is also under control of the molecular clock system (Gil-Lozano et al., 2014). In addition, GLP-1 release by L-cells can also be triggered via different routes not involving bile acid-induced TGR-5 activation (Lim and Brubaker, 2006). These include indirect routes via endocrine and neural signals induced by the presence of food in the stomach and upper intestine (Lim and Brubaker, 2006; Holst, 2007), stimuli thought to be responsible for the rapid postprandial release of GLP-1 (Holst, 2007). When food reaches the ileum, the GLP-1 producing L cells are directly stimulated by glucose, fat (Lim and Brubaker, 2006; Ezcurra et al., 2013), and bile acids (Katsuma et al., 2005; Thomas et al., 2009). The amplitude of the evoked GLP-1 response depends on meal size (Vilsboll et al., 2003). It is difficult to differentiate between the effects induced by GLP-1 in general and the effects that are particularly induced by GLP-1 as a consequence of TGR5 activation by bile acids. Research using TGR5 knockout (*TGR5*^−/−^) mice showed that these mice still produce GLP-1 and seemed not different from wild type mice (Thomas et al., 2009). However, *TGR5*^−/−^ mice fed a HFD displayed impaired glucose tolerance compared to wild types (Thomas et al., 2009). This might indicate that under normal circumstances sufficient GLP-1 is released via signaling routes not involving TGR5-activation. However, this TGR5-independent GLP-1 signal might not be proficient under more extreme circumstances e.g., when high amounts of fat are digested.

### Intestinal GLP-1 signaling to the central nervous system via systemic circulation

Intestinal GLP-1 can reach the brain via two major routes, one being via the systemic circulation and interacting with GLP-1 receptors in the brain (Orskov et al., 1996; Yamamoto et al., 2003) and the other route being through signaling via the vagus nerve (Abbott et al., 2005; Rüttimann et al., 2009). When GLP-1 is released from the basolateral membrane of the L-cells, GLP-1 is taken up by capillaries and transported to the portal vein and subsequently the liver (Holst, 2007). Nonetheless, only a fraction of intestinal GLP-1 reaches the liver in its active form, because the endothelial membranes of the capillaries express the enzyme dipeptidyl peptidase-4 (DPP-4), which degrades GLP-1 rapidly (Holst and Deacon, 2005). Due to the rapid decay only 25% of the intestinal GLP-1 reaches the hepatic portal vein (Holst, 2007). Of this portion only half reaches the systemic circulation via the liver. DDP-4 is also present in plasma, therefore the small amounts of GLP-1 reaching the systemic circulation have a half-life of only 1–2 min (Holst, 2007). In rats, a regular chow meal led to a transient increase in GLP-1 levels in the hepatic portal vein but not in the vena cava, showing that the postprandial GLP-1 increase is not substantial in the systemic circulation (Punjabi et al., 2014). Contrasting, in humans a postprandial increase in plasma GLP-1 levels was evident, lasting several minutes (Vilsbøll et al., 2001; Calanna et al., 2013; Sonne et al., 2014). The human subjects used for plasma GLP-1 measurements (Sonne et al., 2014) also showed a postprandial increase in plasma bile acid levels (Sonne et al., 2016).

The GLP-1 receptor is expressed in various tissues including the CNS (Richards et al., 2014; Cork et al., 2015). GLP-1 is capable of crossing the BBB (Kastin et al., 2002), but it is questionable whether sufficient intact GLP-1 reaches the BBB and other distal tissues to elicit a substantial effect. Therefore, only the area postrema and subfornical area—circumventricular organs—may be plausible brain regions that could gate peripheral GLP-1 signaling via its GLP-1 receptors (Göke et al., 1995; Orskov et al., 1996; Yamamoto et al., 2003). This pathway was established by iv administration of GLP-1 in rats (Orskov et al., 1996; Yamamoto et al., 2003; Punjabi et al., 2014), however, this pathway might not be substantial under normal physiological circumstances when GLP-1 release is triggered by food intake and bile acids.

### Intestinal GLP-1 signaling to the central nervous system via vagal nerve afferents

The other pathway through which intestinal GLP-1 could signal to the CNS is via activation of vagal afferent fibers. These sensory fibers originate in the nodose ganglion and provide terminals into peripheral tissues, including liver tissue (Dardevet et al., 2004, 2005), hepatic portal vein (Balkan and Li, 2000; Vahl et al., 2007), and lamina propria of the intestine (Berthoud et al., 2004; Nakagawa et al., 2004). These terminals express GLP-1 receptors and are therefore responsive to local GLP-1 levels (Holst, 2007). The nodose ganglion projects to the nucleus of the solitary tract (NTS) in the hindbrain (Nakagawa et al., 2004; Holst, 2007). NTS neurons are bidirectional connected with other brain regions, including the hypothalamus (Ricardo and Koh, 1978; van der Kooy et al., 1984). In animal models the signal transmission after ip GLP-1 administration was abolished following subdiaphragmatic vagal deafferentation or after transection of the brainstem-hypothalamic pathway (Abbott et al., 2005; Rüttimann et al., 2009). This established the importance of the vagal-brainstem-hypothalamic pathway for the signal transmission of GLP-1 from the gastrointestinal tract to the CNS. Subsequently, the brainstem and hypothalamus are connected with brain regions involved in autonomic function, metabolic processing, and cognitive and emotional functioning (Rogers et al., 2016). These findings raise the question whether bile acids themselves could directly interact with the vagal nerve and relay a signal to the CNS. However, we have not found reports that show the expression of bile acid receptors on the vagal nerve.

### GLP-1 signaling via the vagal nerve afferents is involved in glucose metabolism and energy homeostasis

Via the vagal-brainstem-hypothalamic pathway peripheral GLP-1 can affect many brain regions and consequently many processes. However, the most studied effects of peripheral GLP-1 are its inhibitory effect on food intake and increased perception of satiety (Tang-Christensen et al., 1996; Turton et al., 1996; Abbott et al., 2005; Talsania et al., 2005; Williams et al., 2006, 2009; Scott and Moran, 2007; Hayes et al., 2008; Rüttimann et al., 2009; Punjabi et al., 2014), which are both believed to be mediated predominantly by the CNS (Turton et al., 1996). These data suggest that vagal nerve terminals in the lamina propria of the intestine are involved in regulating appetite.

Postprandial, the highest GLP-1 concentrations are found in the lamina propria of the intestine and second in the hepatic portal vein (Holst and Deacon, 2005; Holst, 2007). GLP-1 signaling via vagal afferents in the hepatic portal vein does not modulate food intake (Rüttimann et al., 2009), but is involved in modulating glucose metabolism by interacting with hepatoportal glucose sensors (Balkan and Li, 2000; Burcelin et al., 2001; Vahl et al., 2007). This reveals a pathway through which bile acids may be capable to modulate glucose metabolism: TGR5-mediated GLP-1 secretion acting upon hepatoportal glucose sensors. Indeed, *TGR5*^−/−^ mice on a HFD have impaired glucose tolerance and TGR5 over expression in transgenic mice improved glucose tolerance in combination with increased GLP-1 and insulin secretion (Thomas et al., 2009). The above results indicate a differentiation in GLP-1 pathways: glucose homeostasis is mediated via the vagal afferents in the hepatic portal vein and energy homeostasis is mediated via the vagal afferents in the lamina propria (Rüttimann et al., 2009).

Taken together, bile acids in the intestine can signal to the brain by using GLP-1 as an intermediate molecule to activate vagal nerve afferents in the lamia propria and hepatic portal vein that project to the NTS in the hindbrain and subsequently to the hypothalamus. What the exact contribution of bile acids is in the overall GLP-1 signal is difficult to determine, because other nutrient and indirect signals could trigger GLP-1 release.

### GLP-1 released from neurons in the hindbrain

An important consideration is that in addition to GLP-1 release from the intestine, GLP-1 is also produced in the brain. GLP-1 is secreted from a population of preproglucagon (PPG) cells in the NTS and in the intermediate reticular nucleus within the hindbrain (Han et al., 1986; Drucker, 1990; Larsen et al., 1997; Trapp and Cork, 2015). PPG neurons project to a variety of brain regions involved in energy homeostasis and autonomic control including the hypothalamus, thalamus, and amygdala (Merchenthaler et al., 1999; Llewellyn-Smith et al., 2011; Trapp and Cork, 2015). Central GLP-1 signaling is generally linked to energy homeostasis (Cabou and Burcelin, 2011) and glucose metabolism (Sandoval, 2008; Sandoval et al., 2008). The presence of GLP-1-releasing neurons in the brain adds an extra difficulty to elucidating the effects of peripheral GLP-1 in the brain. Electrophysiological findings indicate that PPG cells receive monosynaptic input from vagal afferent fibers (Hisadome et al., 2010). This could mean that peripheral GLP-1 from the intestine could modulate the activity of PPG cells via vagal nerve afferents and consequently stimulate GLP-1 release in the CNS (Hisadome et al., 2010). However, direct evidence is lacking.

### Glucagon-like peptide-2

Together with GLP-1 also glucagon-like peptide-2 (GLP-2) is released from intestinal L-cells in response to nutrients and bile acids. In addition, also in the brain GLP-2 is released from the preproglucagonergic neurons in the brainstem together with GLP-1. GLP-2 acts via its own G protein-coupled receptor, GLP-2R which is mainly expressed in the gastro-intestinal tract and CNS. In the gut, GLP-2 functions in intestinal mucosal health and stimulates nutrient absorption, and in this way influences energy homeostasis (Baldassano et al., 2016). Recently, it has been shown that GLP-2 also stimulates gall bladder filling via GLP-2R and in a TGR5 independent manner in mice (Yusta et al., 2017). In contrast to GLP-1 and the GLP-1R, the functions of GLP-2 and GLP-2R in the brain have not been studied much, but it is thought that GLP-2 has anorexic effects and reduces appetite by activating the GLP-2R in the ARC of the hypothalamus (reviewed in Guan, 2014; Baldassano et al., 2016). However, in humans, peripheral GLP-2 administration had no effect on satiety or food intake (Schmidt et al., 2003; Sørensen et al., 2003). GLP-2 is not an incretin and does not receive as much attention as GLP-1 with respect to research on glucose metabolism and diabetes (Janssen et al., 2013). We are not aware of any studies that investigated the effects of bile acids or postprandial intestinal GLP-2 release on central GLP-2 functioning. The blood half-life of GLP-2 is a few minutes longer than that of GLP-1, but both are efficiently cleared by the kidneys.

## Concluding remarks

In this review we discussed three different pathways via which bile acids could signal to the CNS. In the direct pathway (Figure 3A), bile acids reach the brain via the systemic circulation. In the brain, the machinery for bile acid signaling is present, i.e., receptors able to bind bile acids and transporters to transport bile acids into neurons (Tables 1–3). However, it remains uncertain whether this pathway is substantial under normal physiological circumstances. More research is required to determine whether the postprandial increase in plasma bile acids is also translated into increased bile acid levels in the brain and whether these amounts are sufficient to activate bile acid receptors expressed in the brain. Considering the current information, we believe that this pathway does not exert a prominent route for bile acid signaling to the CNS.

The indirect pathway mediated by FXR-FGF15/19 (Figure 3B) could exert an effect via the CNS through the presence of FGFRs in the brain. FGF15/19 signaling in the brain is associated with energy and glucose homeostasis. However, it is questionable whether the postprandial increase of plasma FGF15/19 is sufficient for substantial signaling in the CNS. We believe that under normal physiological circumstances the peripheral mediated consequences of FGF15/19 signaling exceed the effects that are possibly mediated via the CNS.

The indirect pathway mediated by TGR5-GLP-1 (Figure 3C) can signal to the CNS via two routes, through the systemic circulation and via the vagal nerve. The latter route is the most significant signaling route, because postprandial GLP-1 levels are high in the lamina propria of the intestine and hepatic portal vein, where vagal nerve terminals are present. The vagal nerve signals to the brainstem and subsequently to other brain regions. Via this pathway bile acids could influence glucose and energy homeostasis, among other things. Currently this seems to be the only noteworthy signaling route to the CNS initiated by bile acids under normal physiological circumstances. However, the exact implications of bile acids for this signaling route and their contribution to the whole-body postprandial response remains an interesting subject for future research.

## Author contributions

All authors contributed to the design and concept of the review. KM drafted the manuscript and provided the figures. AK, MS, and HE critically reviewed the manuscript and attributed with important intellectual content. All authors approved the final version of the manuscript and agree to be accountable for all aspects of the work.

### Conflict of interest statement

The authors declare that the research was conducted in the absence of any commercial or financial relationships that could be construed as a potential conflict of interest.
